# Molecular Thumbprints: Biological Signatures That Measure Loss of Identity

**DOI:** 10.3390/biom14101271

**Published:** 2024-10-09

**Authors:** Pallavi R. Devchand, Michael Dicay, John L. Wallace

**Affiliations:** Department of Physiology and Pharmacology, University of Calgary, Calgary, AB T2N 4N1, Canada; mdicay@ucalgary.ca (M.D.); wallacej@ucalgary.ca (J.L.W.)

**Keywords:** resilience, susceptibility, inflammation, anti-inflammatory drugs, clinical signatures

## Abstract

Each life is challenged to adapt to an ever-changing environment with integrity—simply put, to maintain identity. We hypothesize that this mission statement of adaptive homeostasis is particularly poignant in an adaptive response, like inflammation. A maladaptive response of unresolved inflammation can seed chronic disease over a lifetime. We propose the concept of a molecular thumbprint: a biological signature of loss of identity as a measure of incomplete return to homeostasis after an inflammatory response. Over time, personal molecular thumbprints can measure dynamic and precise trajectories to chronic inflammatory diseases and further loss of self to cancer. Why is this important? Because the phenotypes and molecular signatures of established complex inflammatory diseases are a far cry from the root of the complex problem, let alone the initial seed. Understanding the science behind key germinating seeds of disease helps to identify molecular factors of susceptibility, resilience, and early dietary or drug intervention. We pilot this hypothesis in a rat colitis model that is well-established for understanding molecular mechanisms of colonic health, disease, and transition of colitis to cancer.

## 1. Introduction

**Figure 1:**
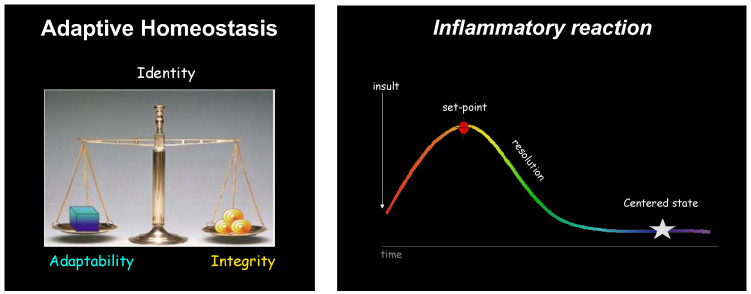
Adaptive homeostasis. Identity is a fine balance between adaptability and integrity. An insult triggers inflammation, which reaches a set point and then resolves successfully to return the organism and tissue back to its centered state (homeostasis).

The body is resilient: not all inflammatory events germinate to disease. In fact, acute inflammation is an adaptive response that is protective, most often goes unnoticed, and is self-resolving (Figure 1).

**Figure 2:**
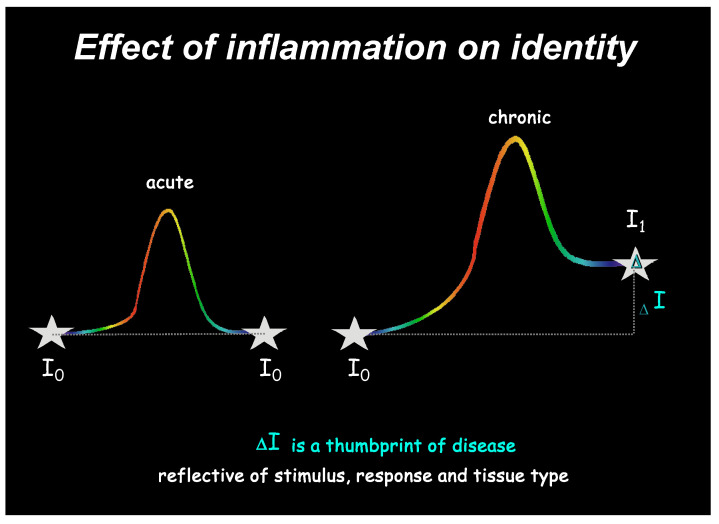
Identity measures a thumbprint of disease. A successful acute inflammation returns the organism back to its original identity **I_0_**. In multifactorial chronic inflammatory diseases, the initial inflammatory response is maladaptive. Incomplete resolution of inflammation returns the organism to a new identity, **I_1_**. The difference in identity, **ΔI**, can be measured and provides a thumbprint of disease.

We provide a preliminary evaluation of our hypothesis that an incomplete response of adaptive homeostasis can be defined by a loss of identity and measured by a biological signature. This biological thumbprint harbors molecular mechanisms that, over time, amplify the further loss of identity. For example, it can seed chronic inflammation and subsequent progression to complex diseases gradually (Figure 2) through successive new identities over time where maladaptation causes I_0_ transitions to I_1_, I_1_ to I_2_, and so on, with many iterations and, in some cases, the transition of I_n_ from chronic disease to cancer.

After a tissue heals from an injury or insult, specialized cells must regain identity to perform their function. Over time, successive losses of molecular determinants of identity can progress to susceptibility to chronic disease, loss of function, and loss of resilience. The definitive case of loss of identity is cancer.

Unresolved inflammation can be quantified by several means and measures, including differences in protein expression, altered metabolic or structural profiles, DNA modifications, and gene expression. The ultimate goal would be detailed and comprehensive biological thumbprints of longitudinal data using current multifactorial and genetic bioinformatic systems. The ambition would be to have biological thumbprints as wholistic clinical measurements with utility in wellness, diagnosis, treatment, and patient responsiveness.

Here, we pilot this hypothesis in a simple, tangible, focused, and well-established model. This rat TNBS-induced colitis model has proven utility in drug discovery and also in understanding molecular mechanisms of colonic health, disease, drug impacts, and transition of colitis to cancer.

## 2. Materials and Methods

Animals: Male Wistar rats (175–200 g) were obtained from Charles River Breeding Farms (Montreal, PQ, Canada). The rats were fed standard laboratory rodent chow and tap water. All experiments were performed as approved by the Animal Care Committee of the University of Calgary (Protocol #M03140) and in accordance with the guidelines of the Canadian Council on Animal Care and NIH.

Induction of colitis using TNBS: Colitis was induced as previously described [1,2]. Briefly, 8 rats were lightly anesthetized with halothane, and an infant feeding tube fitted onto a blunt 18-gauge needle was inserted rectally. The tip of the tube was placed approximately 8 cm into the colon. For treatment, 30 mg of 2,4,6-trinitrobenzene sulphonic acid (TNBS) in 0.5 mL of 50% ethanol was instilled. Control rats were age-matched and naïve (untreated). After 6 weeks, the rats were sacrificed.

In the present study, the severity of colonic damage was blindly scored on a 0 to 10 scale using criteria that have been previously reported in detail [3]. Briefly, a score of 0 represents normal appearance, a score of 1 is given for focal hyperemia but no ulcers, a score of 2 is given for ulceration without associated inflammation, and a score of 3 or greater is given when ulceration and inflammation are both evident (the score increasing further with the extent of ulceration). In most cases, the colonic damage score in rats sacrificed 6 weeks after TNBS administration is 0 or 1. In rare cases, ulceration of the colon is observed. However, as this study is focused on resolved colitis, any rat with a colonic damage score of greater than 1 was excluded from further analysis. After scoring, samples of the distal colon were frozen for subsequent measurement of MPO activity as an index of granulocyte infiltration and gene expression studies below. The standard *t*-test was used for statistical analyses (*p* < 0.05).

Isolation of Total RNA: Frozen colonic tissue was lysed in TRIzol using a hand homogenizer. Total RNA was isolated by the TRIzol method followed by DNase I digestion, as described previously [4]. Contaminating genomic DNA was removed using the Message Clean kit (Genehunter, Nashville, TN, USA). The purified RNA was resuspended in DEPC-treated ddH_2_O, quantitated (OD_260_), and qualitated (OD_260/280_) by UV-spectroscopy, as described in [5].

FDD-PCR: FDD-PCR was performed using a modified protocol of Liang and Pardee (2003) [6]. Briefly, cDNAs were generated from 0.2 mg of RNA using 0.2 μM anchor primer 5′ACGACTCACTATAGGCTTTTTTTTTTTTCA3′ and 20 units of Superscript RT enzyme (GIBCO, Waltham, MA, USA) in a 20 μL reaction containing 1 unit RNasin (Promega, Madison, WI, USA), 25 μM dNTPs, 50 mM Tris pH 8.3, 75 mM KCl, 3 mM MgCl_2_, and 10 mM DTT. After one hour of incubation at 42 °C, reactions were terminated by heat inactivation of the enzyme (72 °C for 15 min).

Complementary DNA fragments were amplified with 0.2 μM each of FITC-conjugated anchor and 1 of 24 arbitrary primers in a 20 μL reaction consisting of 1.5 mM MgCl_2_, 20 μM dNTPs, and 1 unit AmpliTaq enzyme (Boehringer Mannheim, Mannheim, Germany) using the following amplification program: 94 °C for 2 min; 4 cycles (94 °C for 15 s, 50 °C for 30 s, 72 °C for 2 min), 25 cycles (94 °C for 15 s, 60 °C for 30 s, 72 °C for 2 min), and an extension cycle of 72 °C for 7 min.

PCR products were resolved on a denaturing 4.5% polyacrylamide gel (Genomyx LR DNA Sequencer, GeneHunter Core Facility, Nashville, TN, USA) and visualized by fluorography. Bands of interest were excised, reamplified, purified and cloned in PCR-Trap vector. The DNA inserts were sequenced in both directions (Harvard Biopolymer Core Facility, Boston, MA, USA). Sequences were identified by BLAST searches.

Semi-quantitative RT-PCR: Gene changes observed in representative animals used in FDD-PCR were confirmed using standard methods of semi-quantitative RT-PCR. All reactions were performed in a thermocycler (*DNAEngine*, MJResearch) using a heated lid. Poly-A containing primer (50 nmol of PolyT_1 2_-T7 primer) was annealed to 3 μg of RNA, in a total volume of 12 μL, by heating to 70 °C for 10 min followed by quick cooling to 4 °C. These primer–template complexes were used in a 20 μL reverse-transcription (RT) reaction performed in 50 mM Tris pH8.3, 75 mM KCl, 3 mM MgCl_2_, 10 mM DTT, and 0.5 mM dNTPs. Prior to the addition of 200 U of Superscript II Reverse Transcriptase (Gibco), the reaction was incubated at 25 °C for 10 min and 42 °C for 2 min. The RT reaction was carried out at 42 °C for 50 min and terminated by heat inactivation of the enzyme (72 °C for 15 min). The cDNA was purified by digestion of RNA with 2 U of RNase H (37 °C for 20 min), followed by purification on a QIAquick gel extraction kit (Qiagen, Hilden, Germany). Finally, the cDNA was eluted in 50 μL of ddH_2_O and stored at −20 °C until further use. The PCRs (total volume of 50 μL) utilized the Qiagen Master mix, 0.2 mM of each gene-specific primer and 2 μL of the above cDNAs. Reactions were performed in a thermocycler using the following program: denaturation at 94 °C for 2 min, amplification for 30 cycles (94 °C for 0.5 min, 48 °C for 0.5 min, 72 °C for 2 min), followed by extension cycle of 72 °C for 10 min. Annealing temperatures differed depending on the primer sets used. Products were separated by electrophoresis of 10 μL of PCR on 1% agarose gels and visualized by ethidium bromide staining. Results were photographed, scanned, and analyzed for comparative band intensities using the NIH Imager program, described in [5]. As with this pilot, follow-up experiments will continue to provide confirmation of the DD-PCR results by using the tissue/organs from mice that were used to generate the DD-PCR (*n* = 3 expectation of at least of 3 determinates for each sample) and then also in the other mice (*n* = 8) of the two cohorts in the TNBS-colitis experiment described above. The standard *t*-test will be used for statistical analyses.

## 3. Results

### 3.1. Rat Model for a Thumbprint of Unresolved Colitis

Over the past decades, the TNBS rat model has proven useful in understanding the biology of gut health, disease mechanisms, pharmacological intervention, exploration of new investigational drugs, and most notably, in understanding the risks and benefits of over-the-counter medicines, like globally consumed non-steroidal anti-inflammatory drugs. TNBS produces a disease that is histopathologically similar to Crohn’s disease [1]. Key roles for T cells and for several cytokines that have been shown to be important in human IBD have also been shown to be important in the rat TNBS-induced colitis model. This experimental system involves only a single treatment of the rodent (no surgery, etc.) and has been well characterized in terms of the resolution phase of colitis, the boundary to colon cancer and the pharmacology of xenobiotics related to eicosanoid function in the GI, particularly COX- 2-related xenobiotics; (for review, see Wallace and Devchand, 2005 [7]).

This single shot of controlled and localized administration of TNBS is in contrast to the DSS model of colitis, in which the animals are given DSS in drinking water for various periods of time. DSS produces inflammation of the intestine but not very much ulceration. Since our hypothesis deals with post-colitis effects, it is imperative to keep the confounding variables, such as the degree of colitis and site of inflammation, to a minimum. Importantly, transmural inflammation or granulomas (features of Crohn’s disease) are both induced by TNBS.

In previous studies, we found that colitis had resolved by 6 weeks after TNBS administration; that is, the macroscopic appearance of the colon, colonic myeloperoxidase activity (a marker of granulocyte infiltration), and colonic prostaglandin E_2_ synthesis were no longer different from those in healthy controls [1,2].

**Figure 3:**
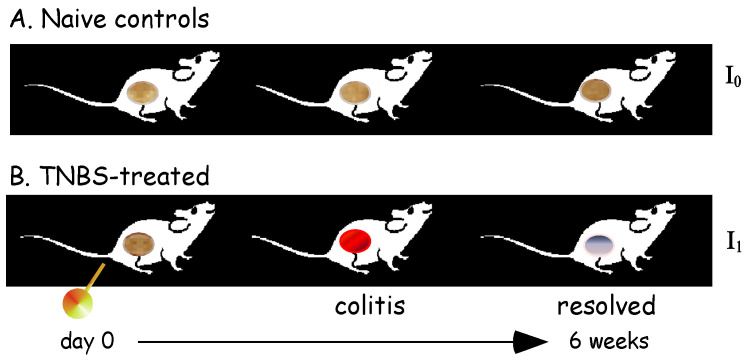
Experimental model. After 6 weeks, TNBS-treated rats (identity **I_1_**) were compared to naïve controls (original identity **I_0_**). Tissues around the precise location of TNBS treatment were harvested for analysis.

Here, the aim of our hypothesis was to compare the difference in the identity of resolved inflammation. Therefore, this simple experiment compared two sets of rats: naïve controls versus post-colitis (Figure 3).

Since the single application of rectally administered TNBS was defined as 8 cm into the colon, the experiment allowed for precision tissue harvesting.

### 3.2. Visualizing Unresolved Inflammation

Here, a preliminary test of our hypothesis was performed using gene expression measurements. In our hands, differential-display polymerase chain reaction (DD-PCR) has been particularly useful in the identification of eicosanoid ligand-triggered pathways [4]. The primary advantage is highly reproducible and tangible data sets at every step of the process. This is often apparent in low-copy number transcripts and alternatively spliced transcripts (often in 3′ non-coding regions). The high-resolution separation of cDNAs on a sequencing gel affords advantages over cDNA CHIP arrays, including identification of novel transcripts, novel alternate spliced transcripts, and low-level transcripts such as nuclear cofactors; for review, see Liang and Pardee, 2003 [6]. Inherent in this method is the cloning of products that correspond to differentially expressed fragments of genes and can subsequently be used as probes in Northern blots, for example. These advantages are particularly important for pathway discovery so that the technique can be streamlined and derivative reagents like clones can be multifunctional for future use (Northern blots, in situ hybridization, FISH). The trade-off on these advantages is time and effort—the high copy number of transcripts that differ in comparison groups could most probably be identified by array or RNAseq analyses in a shorter time frame.

**Figure 4:**
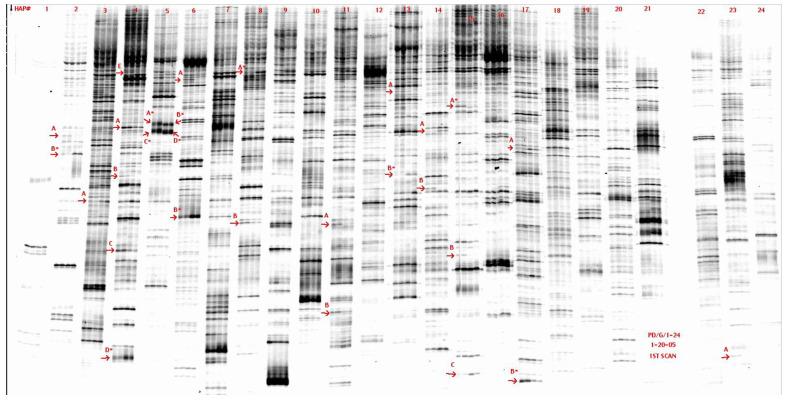
Fluorescent Differential Display Polymerase Chain Reactions. RT-PCR reactions of each treatment group were resolved on a sequencing gel. Lanes derived from each primer pair (1–24) are labeled above, with the left two lanes from naïve controls (original identity **I_0_**) and the right two lanes resolved TNBS-treated rats (identity **I_1_**). The bands of differential expression are indicated and labeled systematically. Original image can be found in Supplementary Materials.

In this pilot experiment, twenty-four cDNA subsets were analyzed for reset after colitis. Duplicate samples of rats of each group were used for gene expression analyses by fluorescent differential display polymerase chain reaction (Figure 4). Close examination of all FDD-PCR reaction lanes revealed that ΔI was very small: ~30 bands differed when comparing naïve rats versus rats that reset after colitis. These differences varied in signal character, including intensity (high or low) and direction of expression (up-regulated or down-regulated).

### 3.3. Tangible Data

**Figure 5:**
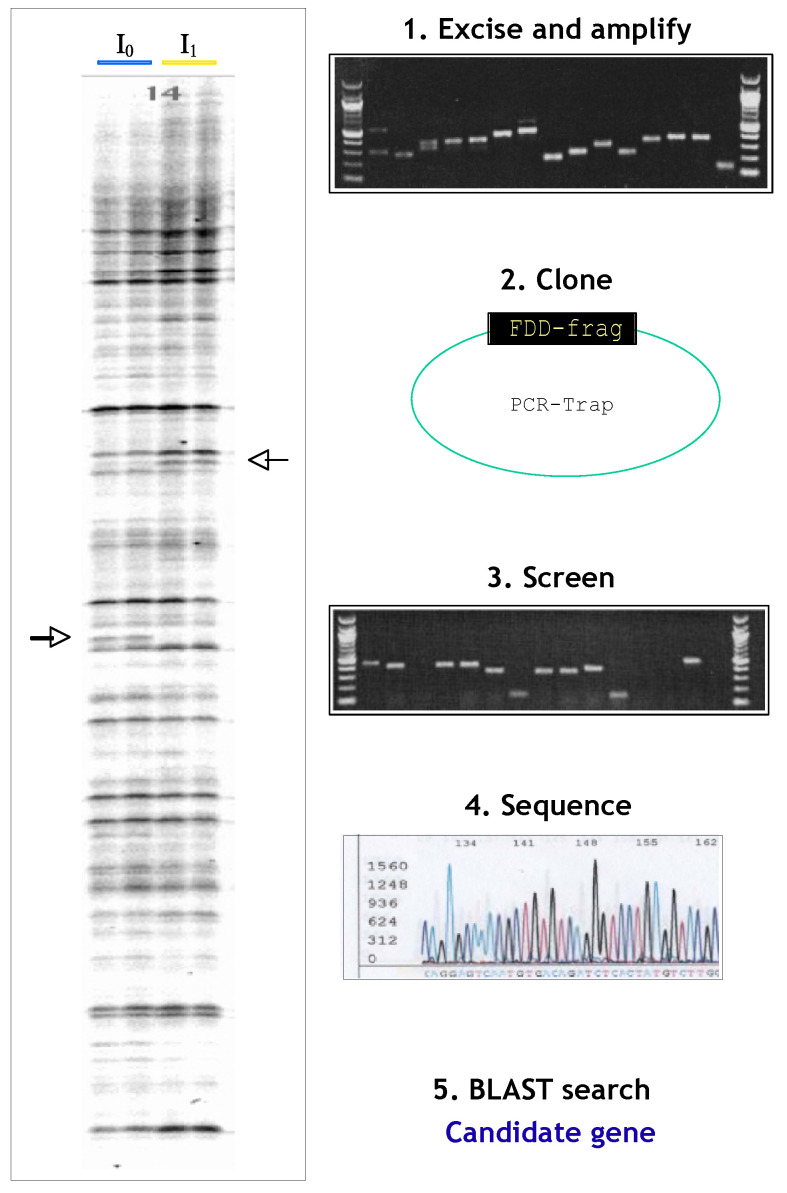
Identifying the candidate genes. This schematic shows the pipeline of analysis. Bands of interest were excised, amplified by PCR, and ligated into the PCR-TRAP vector to derive bacterial clones. After screening, plasmid inserts were sequenced. The candidate genes were identified by BLAST search. Original image can be found in Supplementary Materials.

Twenty bands were excised for further processing (Figure 5). The fragments were amplified, cloned, screened, and sequenced.

### 3.4. BLAST to Gene Identification

Eighteen bands were successfully extracted and cloned. Sequence matches were identified by public database homology using BLAST (Table 1 and Table 2). Referencing back to the original bands in the FDD-PCR sequencing gel, 12 were down-regulated in post-colitis and 6 up-regulated. Table 1 and Table 2 list the genes identified, including *sv2b*, *fra-2*, *sephs-1*, *pallidin*, *DNA primase*, *ptger4*, *rbbp5*, and *mical 3*. Interestingly, in rats with post-resolved colitis, our analysis indicated a potential switch in isoform expression of synaptic vesicle glycoprotein 2: fragment of *sv2b* transcript 1 was up-regulated (Table 2) whereas that of transcript 2 was down-regulated (Table 1). Of note, both *sv2b* transcripts are of low-signal fragments on the FDD-PCR gel. Some excised bands are mapped to chromosome regions or by homology to other species. And one is coded on the mitochondrial genome.

## 4. Discussion

In our hypothesis, we approach health as a challenge to modulate molecular mechanisms of adaptive homeostasis that define the dynamic border of identity. In humans, chronic inflammation of colitis is associated with a predisposition to colorectal cancer, and the risk of incidence increases with the duration of colonic inflammation [8]. This fact supports the concept that chronic inflammation results in small but significant changes in identity, and over time, the increased inability to maintain homeostasis after successive bouts of inflammation, compounded with other environmental triggers, ultimately manifests as the changed identity of cancer.

Here, our hypothesis focused on the final stages of the resolution phase of inflammation to better understand how, after one bout of substantial inflammation, a specialized tissue like the colon retains identity. The reparative ability of the colon is remarkable. At the transcript level, the measured **ΔI**, the change in identity post-colitis, was very small. Only ~30 differences were observed from the 24 subsets of cDNA that were analyzed by FDD-PCR. Sequenced fragments identified several interesting proteins: transcription factors (the fos protein FRA2), the chromatin modulator Rbbp5, DNA maintenance enzyme (DNA primase), vesicle transport and cellular motility (Palladin, the transmembrane transport Svb2, the enzyme Mical3, the hyaluronan-binding protein HMMR), the eicosanoid receptor PTGER4, and the selenide metabolic enzyme SEPHS2.

In the context of our animal model of defining a biological fingerprint post-resolution of TNBS-induced colitis, the identification of PTGER4 is particularly exciting. Altered expression of this eicosanoid G-coupled receptor has long been associated with Crohn’s disease in humans. For example, in a genome-wide association study, Glas et al. (2012) identified a region at Chr 5p13.1 that was associated with both modulating PTGER4 expression and susceptibility to Crohn’s disease [9]. Prager et al. (2014) used a subset of German patients to refine the *rs7720838* variant as a determinant of increased susceptibility to disease [10]. Most recently, Drew et al. (2024) deduced that variants in this 5p13.1 region (like *rs350047*) are mechanistically relevant to the chemo-preventative effect of regular aspirin/non-steroidal anti-inflammatory drug (NSAID) use in patients [11]. Interestingly, using an animal model, Na et al. demonstrated that PTGER4 expression in intestinal macrophages is essential for supporting the regeneration of injured epithelium [12].

**Figure 6:**
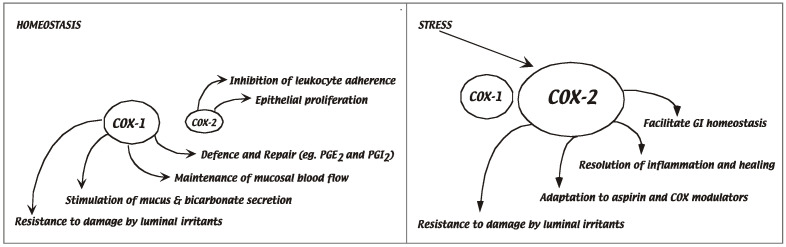
Duality of COX-2 function in dynamics of adaptive homeostasis.

At the molecular level, the mechanisms that cause the disease remain elusive [13]. Current treatments of colitis, like the NSAID mesalamine, aim to control chronic inflammation to sustain colonic integrity over time [14]. Substantial evidence indicates that one target of NSAIDs, the enzyme cyclooxygenase-2 (COX-2), plays several central roles in modulating molecular and cellular mechanisms of gut mucosal defense (Figure 6 and Wallace and Devchand, 2005 [7]). Under normal healthy conditions, COX-2 couples to a battery of downstream enzymes to generate potent lipid mediators involved in processes of adaptive homeostasis, healing, and innate immunity. These include the regulation of mucus and bicarbonate secretion by the gastric and duodenal epithelium, mucosal blood flow, epithelial cell proliferation, epithelial restitution, and mucosal immunocyte function. In the context of an inflammatory reaction, the COX-2 axis is dynamic: in combination with downstream enzymes, it regulates the amplification of the inflammation and also modulates the resolution program in part via prostaglandin D_2_ and E_2_ lipid signaling pathways [3].

Under normal healthy conditions of the gastrointestinal system, endogenous COX-2-derived lipid mediators are involved in processes of adaptive homeostasis, healing, and innate immunity. The COX-2 gene is rapidly induced by stress, and the downstream products are potent lipid signals that enhance resistance to injury and regulate the dynamics of inflammation and resolution.

To date, there are no polymorphisms in the COX-2 gene itself that have been linked to IBD (http://www.ncbi.nlm.nih.gov/projects/SNP, accessed on 12 October 2020). The functions of prostanoids are dynamic, site-specific, and complex [15]. Given the myriad of potent lipid signals and pathways that connect to COX-2 in health and disease states, the duality of COX-2 function in the dynamics of adaptive homeostasis of the GI tract is an important clue to colitis-related disease susceptibility. For instance, COX-2 protein levels remain elevated after a single bout of colitis, and the increased enzyme activity is a determinant of susceptibility to genotoxic-induced cancer formation [16]. It is clear that dysregulation of COX-2 function can be associated with various disease states, including diminished resistance to injury, ulcers, colitis, and colon cancer, see Dubois review [17]. However, the nature of specific downstream lipid products and their signal transduction pathways that mediate associated homeostatic functions remain under-explored.

The most well-characterized products of the COX-2 reaction are the AA-derived prostanoids. Major advances have been made in understanding signaling by arachidonic acid-derived lipid mediators [7,18,19,20]. Eicosanoids are generated in small amounts (nano- to mico-molar quantities) and are rapidly inactivated. Even though these autocoids are short-lived, they are potent stimulators of bioactivity. A retrospective view of eicosanoid research emphasizes the importance of a multidisciplinary approach coupled with the use and development of stable mimetics [21]. For example, distinct biosynthetic enzymes downstream of COX-2 dynamically produce the main lipid mediators linked to the resolution of inflammation: PGE_2_ and PGD_2_ [22,23]. The use of a PGD_2_ synthetic antagonist has implicated PGD_2_ as a determinant of colitis-induced cancer formation [16,24].

Intracellular targets for eicosanoids have been postulated for many years. Recent studies indicate that eicosanoid signaling molecules can directly interact with nuclear receptors (PPARs) to modulate gene expression (e.g., [25,26,27,28]). In the context of PMNs where eicosanoid signals are present intracellularly, these ligand-activated transcription factors have potentially significant roles. Consistent with this notion is the finding that in PPARα knock-out mice, an LTB_4_-mediated inflammatory response is attenuated [28]. The role of PPAR β/δ has been a very active area of research in colon cancer models [17] and the mechanisms underlying chemo-preventative effects of NSAIDs [29].

In the context of shared ligands between cell-surface and nuclear receptors, future molecular modeling and docking studies would complement and advance understanding of drug–protein specificity, mechanisms of action, drug dynamics in detailed binding free energy calculations, and underlying biology based on predictions of the strength and stability of the interactions. A companion study in this collection of articles shows a wonderful example of structural studies on the evaluation of PPAR binding to drugs [30].

Many cell surface receptors also modulate gene expression via non-receptor transcription factors in a tightly controlled fashion. One example of an elaborate system is the activation pathway for the nuclear factor kappa B (NFKB) complex containing FOS and JUN transcription factors [31]. In its active form, NFKB is considered a pro-inflammatory transcription factor, as exemplified by its ability to mediate TNFα-induced transcription of the pro-inflammatory cytokine interleukin1b [32]. In this context, our identification of the fos-related antigen *fra2* as a component of the biological thumbprint is an intriguing result of our pilot experiment. At the transcription factor level, reports suggest that inhibition of NFKB might involve competition either for coactivators or for one of the NFKB subunits (e.g., [33]), and lipid signaling to the nucleus has highlighted roles in paracrine, endocrine, and autocrine pathways [19,34]. Interestingly, the *wnt* signal pathway, most characterized for its association with cancer progression, is dysregulated after a bout of colitis, as measured by increased levels of β-catenin protein in the nucleus—a defect that can be corrected with a selective synthetic COX-2 inhibitor [16]. While counter-regulatory systems in the nucleus have a substantial impact on the adaptive homeostasis of the GI tract, the molecular mechanisms remain elusive.

Of note, in a study that pooled 13 population-based studies, Archambault et al. (2021) sought to identify non-genetic determinants of risk for the incidence of early-onset colorectal cancer [35]. Factors considered in this large analysis included regular use of NSAIDs, red meat intake, educational attainment, and alcohol intake. This study indicates that adaptation to the environment (adaptive homeostasis) and choice of lifestyle can provide a foundation for target identification of those most at risk. Our hypothesis takes a molecular viewpoint of tapping into the early stages of adaptive homeostasis. The results from our pilot experiment support our hypothesis to offer a basis for further study.

We envision the use of biological thumbprints in the clinic and in future research. Some might consider the use of this biological thumbprint over a lifetime to map the bigger picture of aging over time as the body adapts to the environment (e.g., obesity, non-insulin-dependent diabetes). For example, this might be reflected in key metabolites (surrogate markers) combined with personal, holistic parameters in routine physical examinations (weight, blood work, urine test, blood pressure measurements, etc.). Others might consider the use of biological thumbprints simply as maps of the trajectory of unresolved inflammation to complex diseases, such as cardiovascular disease, COPD, Crohn’s disease, and cancer progression. With the rapid advance in computational ability, biological thumbprints could be reflective of autocrine, endocrine, and paracrine systems and measure identities within a tissue, an organ, or an entire body. Simply put, biological thumbprints could be reflective of the dynamics of the health state, measuring progression to disease and also recovery toward the original healthy self.

## 5. Conclusions

Walter Bradford Cannon [36] coined the classical definition of adaptive homeostasis in 1929. Our hypothesis focuses on this definition with the angle that identity is a fine balance of adaptability with integrity. We defined the scientific concept of biological thumbprints that can measure the success of a defined process of adaptive homeostasis and define health states throughout the dynamic trajectory over a lifetime.

Here, we have used the adaptive response of inflammation as an example. A successful acute inflammation returns the organism back to its original identity **I_0_**. In multifactorial chronic inflammatory diseases, the initial inflammatory response is maladaptive. Incomplete resolution of inflammation returns the organism to a new identity, **I_1_**. The difference in identity, **ΔI**, can be measured and provides a thumbprint of disease.

We envision that molecular thumbprints would be particularly useful in complex inflammatory diseases. The progressive approach of our hypothesis helps create personalized maps of health states with potential cost-effective use in the clinic. In a more general sense, these thumbprints would be a measure of wellness. How do these wellness measures synchronize and synergize to maintain health over a lifetime? It’s complex. It’s personal. It is tangible.

## Figures and Tables

**Table 1:** Down-regulated genes. These 12 cDNA fragments showed lower expression in rats with resolved colitis. For each, the upper number indicates location on the FDD-PCR gel. Gene names identified by BLAST are in bold. The protein name, when available, is indicated. Finally, the corresponding regions of the transcript that were excised and amplified from the band are indicated.

**Low Signal**	**High Signal**
**2A-1** **Sv2b** Synaptic vesicle glycoprotein 2b Transcript 1 5271-5872	**5A-1** **Pallidin** Syntaxin-13 interacting protein 1871-2041 1770-1846
**14B-3** Sim to region on Chr4 on mouse 76075-75953	**5A’-1** Aligns to mChr8 BAC clone Possibly a MHC gene
**23A-1** **Fra-2** Fos-related antigen DNA 105-234	**5C-2** Rat homologue of mKIAA1930 4091-4386
**4B-1** **Sephs 1** Selenophosphate synthetase	**6A-1** **DNA Primase** Subunit 49 1171-1374 1407-1591
**15B-2** Homology to region in mChr1	**11A-1** **Ptger** EP4 receptor 2619-2811
	**15A-4** Sim to region on Chr11 on mouse 63633-751. 63766-882
	**21B-2** **Rbbp5** Retinoblastoma binding protein 5 Similar to mChr1 23480-23132 in rat 766-897;898-970

**Table 2:** Up-regulated genes. These 6 transcript fragments showed higher expression in rats with resolved colitis. For each, the upper number indicates location on the FDD-PCR gel. Gene names identified by BLAST are in bold. The protein name, when available, is indicated. Finally the corresponding regions of the transcript that were excised and amplified from the band are indicated.

**Low Signal**	**High Signal**
**2B-w** **Sv2b** Synaptic vesicle glycoprotein 2b Transcript 2 5271-5942	**5D-1** Rat homologue of *m.musc* cDNA BC051083 1095-1329
**21A-2** Similar to region on Chr 15 on mouse 168293-7953	**8A-1** Rat homologue of **MICAL3** Microtubule-associated monooxygenase calponin Lim-domain containing 3 1546-1694
	**13B-1** Receptor for hyaluron-mediated motility Coded on mitochrondrial genome 308-144
	**14A-4** Image clone 7123360 2688-2770 2519-2631 78bp homologous to interferon-induced P44

## Data Availability

All data obtained are presented in the manuscript.

## Supplementary Materials

The following supporting information can be downloaded at: https://www.mdpi.com/article/10.3390/biom14101271/s1, Original images of Figure 4 and Figure 5.
