# Supplementary figures and images for: Molecular Thumbprints: Biological Signatures That Measure Loss of Identity
Source: Biomolecules. 2024 Oct 9;14(10):1271. doi: 10.3390/biom14101271 (PMC11506567; doi:10.3390/biom14101271)

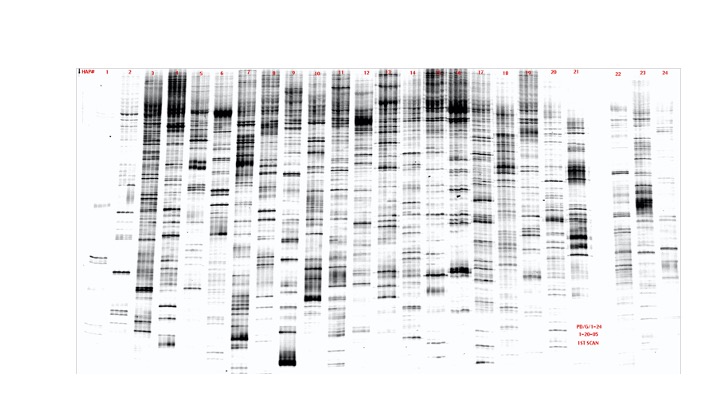

Supplement: Supplementary file 1 [file biomolecules-14-01271-s001.zip › Figure 4.jpeg]

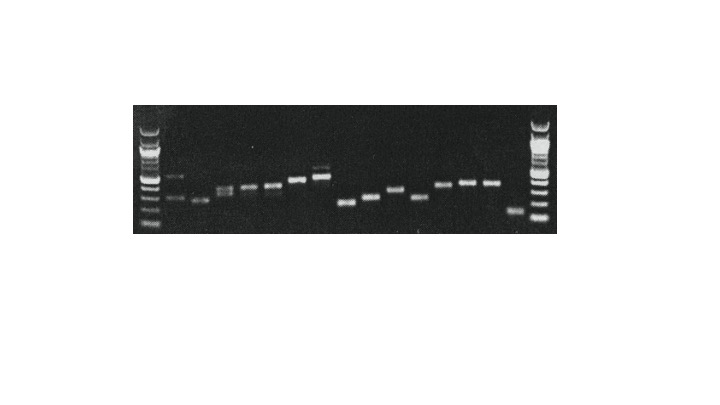

Supplement: Supplementary file 1 [file biomolecules-14-01271-s001.zip › Figure 5_1.jpeg]

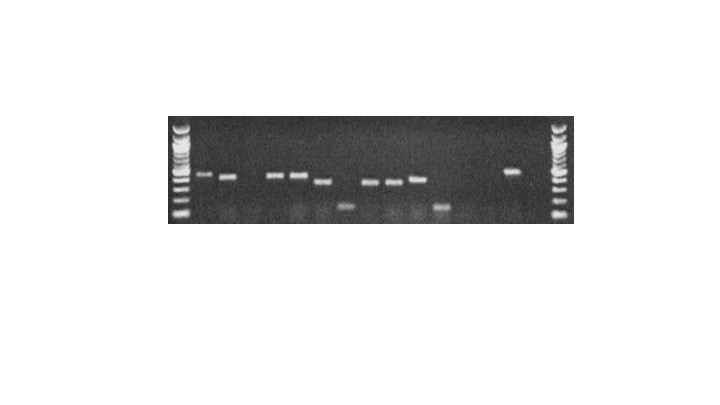

Supplement: Supplementary file 1 [file biomolecules-14-01271-s001.zip › Figure 5_3.jpeg]
